# Social and non-social feedback stimuli lead to comparable levels of reward learning and reward responsiveness in an online probabilistic reward task

**DOI:** 10.3758/s13428-023-02255-6

**Published:** 2023-10-16

**Authors:** Uta Sailer, Franz Wurm, Daniela M. Pfabigan

**Affiliations:** 1 Department of Behavioural Medicine, Institute of Basic Medical Sciences, Faculty of Medicine, University of Oslo, Sognsvannsveien 9, 0372 Oslo, Norway; 2 Department of Psychology, Leiden University, Leiden, 2333 AK The Netherlands; 3 Leiden Institute for Brain and Cognition, Leiden, 2333 AK The Netherlands; 4 Department of Biological and Medical Psychology, Faculty of Psychology, University of Bergen, Jonas Lies vei 91, 5009 Bergen, Norway

**Keywords:** Drift diffusion modelling, Reward, Social feedback, Processing advantage, Decision making

## Abstract

Social stimuli seem to be processed more easily and efficiently than non-social stimuli. The current study tested whether social feedback stimuli improve reward learning in a probabilistic reward task (PRT), in which one response option is usually rewarded more often than the other via presentation of non-social reward stimuli. In a pre-registered online study with 305 participants, 75 participants were presented with a non-social feedback stimulus (a star) and information about gains, which is typically used in published PRT studies. Three other groups (with 73–82 participants each) were presented with one of three social feedback stimuli: verbal praise, an attractive happy face, or a “thumbs up”-picture. The data were analysed based on classical signal detection theory, drift diffusion modelling, and Bayesian analyses of null effects. All PRT variants yielded the expected behavioural preference for the more frequently rewarded response. There was no processing advantage of social over non-social feedback stimuli. Bayesian analyses further supported the observation that social feedback stimuli neither increased nor decreased behavioural preferences in the PRT. The current findings suggest that the PRT is a robust experimental paradigm independent of the applied feedback stimuli. They also suggest that the occurrence of a processing advantage for social feedback stimuli is dependent on the experimental task and design.

## Introduction

Our daily lives are filled with social feedback signals – the nod of a teacher encouraging a student to speak up, the smile of a conversation partner signalling continued interest, or the aversion of one’s gaze aimed at stopping another person’s approach. Individuals make use of these signals to implement adaptive changes in their behaviour, hoping for favourable outcomes. Although these social feedback signals are ubiquitous in daily life, psychological research has mostly used non-social signals in feedback and learning experiments in the past decades. Non-social feedback stimuli allow for a high degree of experimental control but lack ecological validity. Tackling this problem, the last years have seen an increasing interest in establishing more realistic experimental settings with “social” stimuli to signal performance and reward feedback.

A recent review by Matyjek et al. (2020) characterised the different dimensions of social and non-social feedback stimuli, and also pointed at potential problems when directly comparing them. For example, the most common comparisons in the literature are conducted between monetary stimuli (as a non-social example) and facial displays (as a social example) serving as feedback signals. These comparisons are often confounded by several factors: reinforcer type (primary/innate [faces] vs. secondary/learned [money] reinforcers), temporal proximity of delivery (immediate [faces] vs. delayed [money] delivery), duration (longer lasting [money] vs. transient [faces]), tangibility (abstract vs. touchable), and naturalness (symbolic vs. naturalistic stimulus depictions) (Matyjek et al., 2020). These are just a few stimulus dimensions that potentially add to reported processing differences between monetary and facial feedback stimuli (e.g., Flores et al., 2015; Rademacher et al., 2010; Spreckelmeyer et al., 2009). Along these lines, a study using a probabilistic reward-based decision-making task (PRT; Pizzagalli et al., 2005) found faster reaction times and higher accuracy with monetary than facial feedback stimuli (Pechtel et al., 2013). Notably, the study design did not control for the fact that participation in one group was incentivised with an additional monetary bonus that was missing in the other group. Other studies did use social and non-social stimuli in this probabilistic reward task, but did not compare their effects with each other (Chevallier et al., 2016), or statistically eliminated the impact of monetary vs. social praise feedback on task outcomes (Janes et al., 2015).

Attempting to limit the influence of several confounding stimulus dimensions, our own research investigating social and non-social feedback stimuli used both naturalistic depictions and line drawings of thumbs up/down as social feedback stimuli and compared those to plus/minus symbols matched on visual complexity (Pfabigan et al., 2019; Pfabigan & Han, 2019). This approach ameliorated differences in temporal proximity of delivery, duration, and tangibility, and partly those of naturalness. By extracting event-related potentials and oscillatory activity during performance feedback, we observed neural and behavioural processing advantages for social feedback stimuli compared to non-social ones (Pfabigan & Han, 2019). Pioneering psychological studies (Allport, 1920; Dashiell, 1930; Gates & Rissland, 1923; Zajonc, 1965) as well as more recent research (Hurlemann et al., 2010) seem to support such a processing advantage of social over non-social feedback stimuli. However, the underlying mechanisms of this processing advantage are not well understood, and therefore it is unclear how social stimuli could lead to observed behavioural improvements. This is of crucial importance as the use of social feedback stimuli could be a simple strategy to improve behaviour in learning settings.

Within the current study, we investigated two potential mechanisms that might contribute to the proposed processing advantage of social stimuli. First, it might be possible that the use of social feedback stimuli leads to an increase in one’s ability to learn from positive reinforcement (i.e., reward learning). Second, it might be possible that the use of social feedback stimuli acts on reward responsivity in general. The latter suggestion can be investigated by disentangling sub-processes of the decision-making process that influence reward responsivity via computational modelling. For example, the use of social feedback stimuli might increase the speed of evidence accumulation (i.e., the drift rate) on a trial-by-trial basis (Gold & Shadlen, 2007), or result in a larger response bias towards the rewarded response option (i.e., starting bias, Ratcliff et al., 2016). The unique combination of stimulus dimensions (e.g., primary reinforcer, immediate reward, natural stimuli) of social stimuli might account for this biased behaviour. It could therefore be that the use of social feedback stimuli increases the learning rate of rewarded choice options, influences evidence accumulation (drift rate), or biases behavioural responses towards the rewarded choice option (starting bias), or a combination of all these possibilities.

To test whether reward learning or sub-processes influencing reward responsivity, or their combination, underlie the proposed processing advantage of social feedback stimuli, we conducted a pre-registered online experiment. Reward learning and sub-processes of reward responsivity were assessed with the probabilistic reward task (PRT; Pizzagalli et al., 2005). This task is framed as a visual discrimination task in which correct choices are sometimes followed by positive reinforcement. Unbeknownst to the participants, one response option is rewarded three times more often than the alternative one, which usually introduces a preference (i.e., response bias) for the rewarded response option in healthy individuals. One participant group was presented with a non-social feedback stimulus as reward, while three other groups were presented with different instances of social feedback stimuli as rewards. We capitalised on the literature that consistently reported such a response bias as an objective measure of reward learning (Pizzagalli et al., 2005), as well as more recent computational modelling approaches of the PRT to assess latent parameters such as drift rate and starting bias (Eikemo et al., 2017, 2019; Huys et al., 2013).

In line with our previous research (Pfabigan et al., 2019; Pfabigan & Han, 2019), we hypothesised that reward learning and reward responsivity would be higher when using social compared to non-social feedback stimuli (preregistered Hypothesis 2). Additionally, we explored whether trait reward responsiveness and hedonic capacity were associated with PRT-derived response bias and computational modelling parameters in the whole sample.

## Materials and methods

### Participants

In our pre-registration, we reported an *a priori* sample size calculation with a target sample size of 304 participants calculated with G*Power (Faul et al., 2007). However, we later noticed that the program is not suitable for these mixed design calculations. Using the program PANGEA (Westfall, 2016), we now report the minimal effect sizes of interest observable in the current study for our main hypotheses. With a four-level between-subject factor (feedback type) and a three-level within-subject factor (experimental block) and at least 73 participants in each group, this study has 80% power to detect a main effect of feedback type with d = 0.33, 81% power to detect a main effect of experimental block with d = 0.27, and 80% power to detect an interaction effect of feedback type by experimental block with d = 0.38.

Via an online participant recruitment platform, 327 English-speaking volunteers aged between 18 and 55 years were recruited. Twenty-two datasets were excluded from further analyses because participants failed to conform to task instructions (i.e., had an accuracy rate of below 50% or used only one response button for more than 75% of the trials). The final sample consisted of 305 participants with a mean age of 26 years (SD = 7.54): 138 women, 166 men, and one participant who preferred to not disclose their sex. The experiment was presented in four parallel versions (i.e., feedback-type groups) which were posted online in a mixed order to recruit approximately 40 participants each per online batch. Participants gave online consent before participation. The study was conducted in accordance with the 1964 Declaration of Helsinki and approved by the Regional Committee for Medical Research Ethics South East Norway (REK Sør-Øst B, project 26699), and by the Norwegian Centre for Research Data (NSD) to comply with GDPR regulations. The study was preregistered on the Open Science Forum (OSF) before data collection; anonymized raw data and analyses scripts are provided on the OSF and GitHub (see Data Availability statement). This online study is part of a larger research project investigating the effects of gut hormones on reward processing (see https://osf.io/f9rkq) and was conducted as a side project during a COVID-19 lockdown when the larger project was on hold. Due to the context of the larger project, this study preregistered also a hypothesis addressing whether reward responsiveness and learning would be associated with subjective hunger feelings (preregistered Hypothesis 1). Hence, subjective ratings of bodily states and demographic information such as on height and weight were assessed to link results of the online experimental task to the larger project on gut hormones.

### Online recruitment

Participants signed up for the experiment via the platform https://prolific.co. After providing consent, demographic variables were assessed. Afterwards, subjective ratings and psychological questionnaires were filled in via an online survey tool (https://nettskjema.no/ [a survey solution developed and hosted by the University of Oslo (nettskjema@usit.uio.no)]), storing participants’ responses on an encrypted server. After filling in all questionnaires, participants used a link to access the experimental task, which was hosted on the website https://pavlovia.org/. At the end of the experimental task, participants were re-directed to https://prolific.co, where their task completion was recorded. The questionnaire part took about 15–20 min to fill out; the subsequent experimental task took about 20 min. Each participant received £5.63 for study completion.

### Experimental task

**Fig. 1:**
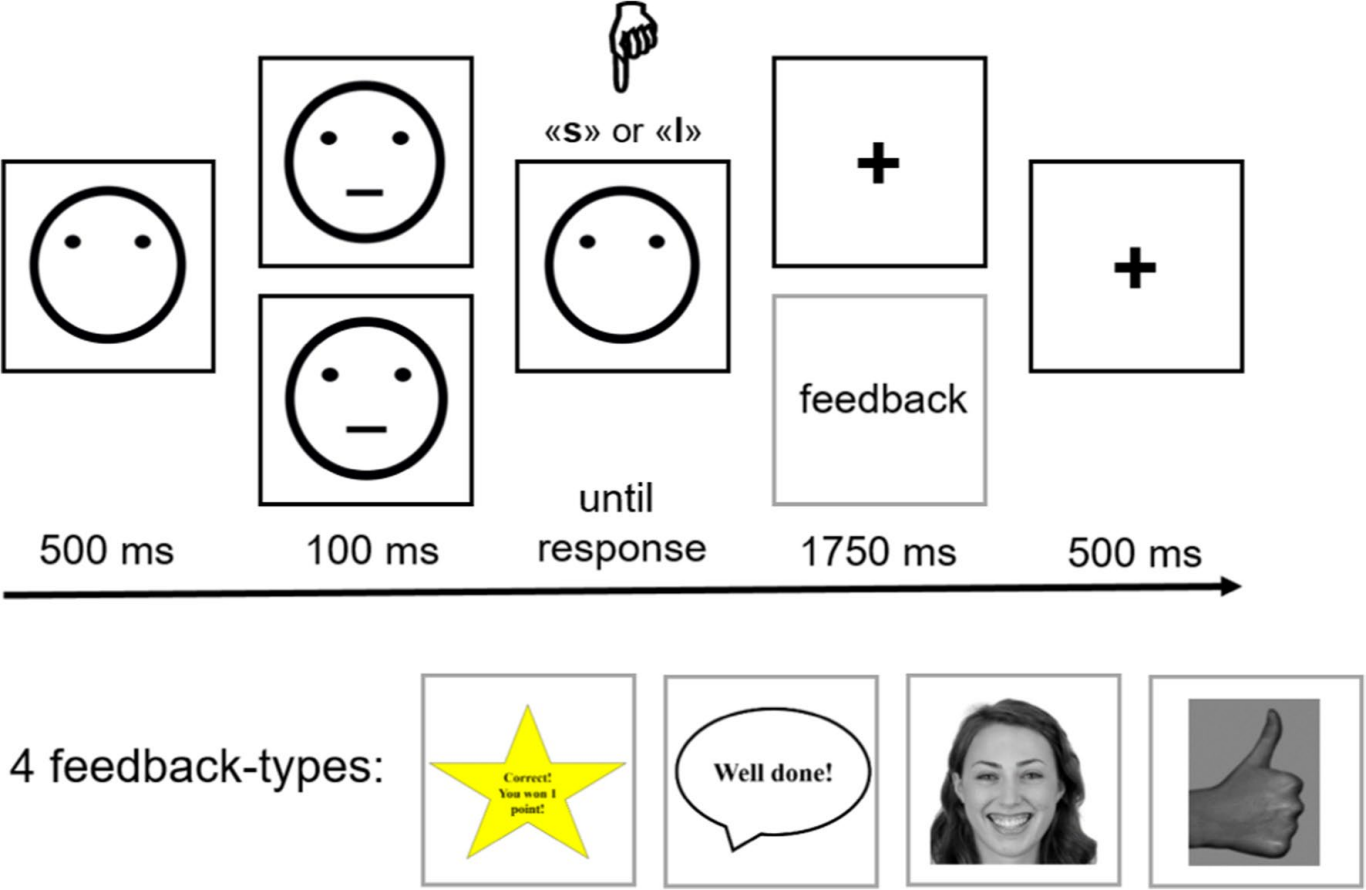
Trial timing of the PRT and the four feedback-type groups

Participants performed an adapted version of the probabilistic reward task (PRT; Pizzagalli et al., 2005) to assess their reward responsiveness. The PRT is framed as a perceptual decision task in which participants have to make a choice between two visually similar options. Skewed reward schedules are used in the task to introduce a response bias, which is supposed to reflect the propensity of behavioural change as a function of available rewards. Each trial starts with the presentation of a mouthless cartoon face for 500 ms (see Fig. 1). Subsequently, a line is blended in for 100 ms constituting the mouth of the schematic face. Afterwards, participants have to indicate via button press whether the mouth was long or short (by pressing buttons ‘s’ for short or ‘l’ for long with their index fingers on a keyboard). There was no response time limit. On selected trials, participants were presented with a feedback stimulus for a duration of 1750 ms; on the remaining trials a fixation cross was presented for the same duration. During the inter-trial-interval, a fixation cross was displayed for 500 ms until the next trial started. The visual stimuli differ only minimally from each other – by 1 mm in the laboratory task version. Thus, in combination with the short presentation duration, the correct identification of the mouth length is challenging. Accuracy rates are about 75% in laboratory PRT versions (Eikemo et al., 2019).

At the beginning of the experiment, participants were informed that they will receive feedback after a correct answer in a few cases, and that they will be provided with information at the end of the experiment on how often they had received this feedback. This was done to match laboratory task versions in which participants received information of how much money they had won at the end of the task. Unbeknownst to the participants, 75% of all correct responses were rewarded with positive feedback for one of the stimuli (i.e., the *rich* response option). For the other stimulus, only 25% of all correct responses were rewarded with positive feedback (i.e., the *lean* response option). The presentation order of rich and lean stimuli was randomised. The assignment of short and long mouths serving as rich and lean stimuli was counterbalanced across participants.

In a between-subject design, four different types of feedback stimuli were presented to induce a response bias towards the rich option. In reference to the original task version, one group of participants saw a yellow star with the information “You have won 1 point!” (Pizzagalli et al., 2005). This group was considered as the non-social feedback-type group. A second group received verbal feedback “Well done!” displayed in a speech balloon (e.g., Janes et al., 2015; van der Veen et al., 2011). A third group was presented with the smiling face of a young woman, taken from the Chicago Face Database (Ma et al., 2015). The last group was presented with a photograph of a realistic hand with thumbs-up, as used in our previous studies (Pfabigan et al., 2019; Pfabigan & Han, 2019). Those three groups were considered as social feedback type groups. A between-subjects design was chosen to avoid potential carry-over effects due to tiredness or a lack of novelty, but also to assess the robustness of the PRT with respect to different feedback stimuli. All visual task displays were programmed for a 1600 × 1200 display resolution. It was not possible to present the stimuli in exactly this display size in the online browser version. Instead, a match-to-desktop resolution approach was chosen which kept the ratio between height and width of the original display size constant. Experimental participation was only possible on laptops and desktop computers.

Participants performed ten training trials with performance feedback (correct/incorrect) once in the beginning. None of the participants was rejected based on their performance in the training. Afterwards, three blocks of 96 trials each were presented. Participants could take breaks in between the blocks. After the third block, participants were informed about how often they had received positive feedback overall. Moreover, participants were asked whether they thought that the short mouth face or the long mouth face was rewarded more often than the other one. This question was asked to assess whether participants had been aware of the skewed reward schedule or not. Further, participants were asked how rewarding they had experienced the positive feedback stimulus (on a nine-point Likert scale from 1 = not at all rewarding to 9 = extremely rewarding) to assess whether the stimuli differed in experienced reward. The current task version was programmed in PsychoPy v2020.1.3 (Peirce et al., 2019) and hosted online on https://pavlovia.org.

### Subjective ratings and questionnaires

All participants provided information about their sex, body weight and height, education level, and whether they were smokers. In addition, female participants were asked to indicate whether they were taking hormonal contraceptives, and whether they were currently in the first week of their menstrual cycle. This last information was collected because previous studies suggested that reward responsiveness could vary in relation to hormonal changes within the menstrual cycle (Diekhof & Ratnayake, 2016). Participants’ ethnicity or cultural identification were not assessed.

To assess current affective state that could potentially influence PRT performance, participants filled in the Positive and Negative Schedule (PANAS, Watson et al., 1988). Twenty adjectives describing either positive or negative affective states had to be rated on a five-point Likert scale, ranging from 1 = very slightly or not at all to 5 = extremely. To assess trait reward responsiveness, participants filled in the Temporal Experience of Pleasure Scale (TEPS, Gard et al., 2006). The questionnaire consists of 18 statements describing joyful situations (e.g., *The smell of freshly cut grass is enjoyable to me.*). Participants had to indicate how true each statement was for them in general, using a six-point Likert scale ranging from 1 = very false for me to 6 = very true for me. To assess hedonic capacity, the Snaith-Hamilton Pleasure Scale (SHAPS, Snaith et al., 1995) was administered. This 14-item questionnaire aims to assess participants’ ability to experience pleasure in the last few days. Participants had to indicate their agreement with statements such as *I have found pleasure in small things, e.g., bright sunny day, a telephone call from a friend* via four options (strongly disagree/disagree/agree/strongly or definitely agree). The analysis scheme by Franken et al. (2007) was used. Additionally, participants rated subjective bodily states and trait interoceptive abilities (see preregistration), which will be reported elsewhere.

### Statistical analyses

#### Pre-registered analyses

Data were analysed with SPSS 26.0 (IBM), jamovi (The jamovi project, 2023; JASP (JASP Team (2022), and MATLAB (MathWorks). To describe the four feedback-type groups, self-reported sex, contraceptive use, current menstruation, education, and smoking status were compared with chi-square tests. Questionnaires and rating data across the four feedback-type groups were compared with one-way ANOVAs after testing for equal variances across groups with a Levene test. Multilevel modelling was conducted to test for feedback-type group differences in reaction times, accuracy rates, and the signal detection theory indices (response bias log b, discriminability log d).

Participants were excluded from analyses if their overall accuracy was below 50% and/or if they responded with only one response button in more than 75% of all trials. This resulted in the exclusion of 22 participants from both the PRT and the questionnaire data. In the remaining participants, trials with response times < 250 ms and > 2500 ms were excluded prior to analyses (approx. 9.9 % overall), based on previous literature (Eikemo et al., 2019).

Single-trial reaction time data were first subjected to a log10 transformation to approximate a Gaussian data distribution. These logarithmized reaction time data were modelled as a function of *feedback type* (comparing the non-social group with each of the three social feedback groups), *block* (comparing blocks 1 vs. 2, and 2 vs. 3), *reward contingency* (effect coding: lean = – 1, rich = 1), and mean-centred *trial number* as continuous predictor, and the interactions *feedback-type x reward contingency* and *block x trial number* as fixed effects. Trial number was included as continuous predictor because we were interested in whether reaction times would vary over the course of the experiment, and over the three experimental blocks. The random effects structure included a random intercept for participant and random slopes for block and reward contingency (model: reaction times ~ 1 + feedback-type + block + reward contingency + trial number + feedback-type:reward contingency + block:trial number + (1 + block + reward contingency│participant). Accuracy rates (% based on valid trials) were subjected to an arcsine transformation to approximate a Gaussian data distribution before they were modelled with *feedback-type*, *block*, and *reward contingency*, and all possible interaction terms as fixed effects. The random effects structure included a random intercept for participant and random slopes for block and reward contingency (model: accuracy ~  1 + feedback-type + block + reward contingency + feedback-type:block + feedback-type:reward contingency + block:reward contingency + feedback-type:block:reward contingency + (1 + block + reward contingency│participant). In line with previous PRT studies, we calculated a response bias (log b) and a discriminability (d’) index in reference to established procedures from signal detection theory (Pizzagalli et al., 2008). The value 0.5 was added to each cell to avoid division by zero. Log b is considered an index of reward sensitivity and reward learning, while log d (or d’) is an index of stimulus discriminability (Pizzagalli et al., 2005). A winsorization procedure was applied for the two indices per group because outliers were detected for each index (Starkings, 2012). Winsorized log b and log d values were separately modelled with *feedback-type*, *block,* and their interaction as fixed effects. The random effects structure included a random intercept for participant (model: log b/log d ~ 1 + feedback-type + block + feedback-type:block + (1│participant). For all multilevel analyses, we used the Satterthwaite method for approximation of degrees of freedom and applied a restricted maximum likelihood estimation for fixed effects. Equal random effects covariance structure was assumed across the four feedback-type groups in all analyses. As effect size measures, semi-partial *R*^2^ is reported (Edwards et al., 2008). Values of 0.02, 0.13, and 0.26 denote small, medium, and large effects (Cohen, 1992).

##### Computational modelling

To assess whether social feedback impacts reward responsiveness, the choice and reaction time data were fitted with a drift diffusion model (DDM). DDMs are a family of sequential sampling models that allow the extraction of information about underlying mechanisms above and beyond descriptive and signal detection approaches (Ratcliff, 1978).

The central assumption underlying the drift diffusion model is that agents continuously gather evidence for their available options when faced with a decision-making problem. Only once a decision threshold in favour of any alternative is reached, i.e., enough evidence is sampled for that option, a corresponding motor response is initiated. Computationally, this idea can be formalised as a sequential sampling process with four parameters. The drift rate parameter (*v*) specifies the evidence generation/sampling, i.e., the speed of evidence accumulation. The boundary separation parameter (*a*) quantifies the amount of evidence that is sufficient for the agent to make a decision. Increases in boundary separation therefore lead to more accurate but slower choices. In contrast, increases in drift rate lead to both more accurate and faster choices. The starting point parameter (*z*) reflects a bias towards one of the decision boundaries, i.e., more frequent and faster choices for the respective options. Finally, the non-decision time parameter (*t*) indicates the temporal offset between choice onset and the start of evidence accumulation.

The model fitting was performed using the rstan package (Stan development team; Carpenter et al., 2017) in R (R Core Team (2022). Parameters were estimated for each individual and block separately by looping through each participant’s trials, assuming a distribution according to the Wiener-first-passage-time. To ensure mutually constrained and reliable estimates, the individual parameters were drawn from corresponding group-level distributions separately for each feedback type and block. The parameters for the group-level distributions were drawn from pre-specified priors. On the group level, the boundary separation, non-decision time and starting bias parameters for each block and feedback type were drawn from uniform distributions. The boundary separation was initialized between 0.1 and 10. The non-decision time was initialised between 0.1 and 1. The starting bias was initialized between – 4 and 4. The group-level drift rate parameters were drawn from normal distributions (initialised as μ = 0 and δ = 10). The respective standard deviation priors were drawn from Cauchy distributions (initialised as μ = 0 and δ = 2.5). On the individual level, all parameters were drawn from a normal distribution with the mean and standard deviations initialized as the corresponding group-level priors. As we were interested in the effect of social and non-social feedback on any of the above mentioned DDM parameters, the drift rate, boundary separation, and non-decision time parameters were estimated separately for rich and lean trials. In line with previous research (Eikemo et al., 2017, 2019), the starting bias for lean trials was defined as the complementary to the starting bias for rich trials, i.e., z(lean) = 1–z(rich). To ensure meaningful sampling, each parameter was additionally constrained to a specific range, effectively truncating the distributions. Boundary separation was limited to be positive (truncated at 0), non-decision time was bound between 0 and 1, and starting bias was bound between – 4 and 4. Please note that the starting bias was mapped from [– 4, 4] to [0, 1] for the Wiener-first-time passage function, using the cumulative density function of the normal distribution (phi). All standard deviation priors were bound to be positive. The hierarchical model was run with four chains with 2000 burn-in samples and 2000 posterior samples each. All chains converged successfully, as indicated by R-hats between 1.000 and 1.002 (Gelman & Rubin, 1992).

For feedback-type group comparisons, the estimated model parameters for drift rate, boundary separation and non-decision time were extracted for each participant and compared across conditions with multilevel models analogous to the ones for reaction times, accuracy, and response bias/discriminability indices. Again, a winsorization procedure was applied per group because of outlier values. Winsorized DDM parameter were separately modelled with *feedback-type*, *block,* and *reward contingency,* and all possible interactions as fixed effects. The random effects structure included a random intercept for participant and random slopes for block and reward contingency when model convergence allowed for those two (model: DDM parameters ~ 1 + feedback-type + block + reward contingency + feedback-type:block + feedback-type:reward contingency + block:reward contingency + feedback-type:block:reward contingency + (1 + block + reward contingency│participant). For the starting bias parameter, the factor *reward contingency* was dropped, as starting bias for lean trials is defined as the complement of the starting bias for rich trials (see above).

#### Exploratory analyses

We calculated the difference between log b in block 3 and block 1 per participant to capture reward learning (e.g., Santesso et al., 2008), and submitted these values to a one-way ANOVA to test for differences between the feedback types. Furthermore, scores on questionnaire scales assessing current affective state (PANAS), trait reward responsiveness (TEPS), and hedonic capacity (SHAPS) were added to the multilevel models of reward bias log b and the DDM model parameters as continuous predictors to explore their potential influence. For the respective mean-centred scores, a main effect as well as the interaction with feedback type were modelled. Further, effects of sex and age were explored. These exploratory analyses were corrected for multiple comparisons (Bonferroni correction for five covariates per dependent variable: p_corr_ < .010). To substantiate null-findings of feedback type in the main outcomes, we report Bayes factors in support for the null hypothesis for simplified statistical models (by collapsing across rich and lean stimuli and the three blocks).

### Results

The four feedback-type groups did not differ significantly from each other in terms of demographics, all *p* values > .182, see Table 1)

**Table 1:** Sample characteristics

		Star	Verbal FB	Happy face	Thumbs up	Total sample	Chi-square test
		*n = 75*	*n = 75*	*n = 73*	*n = 82*	*n = 305*	*p*
Sex	Man	45	37	39	45	166	0.606
	Woman	30	38	34	36	138	
	Prefer not to answer	0	0	0	1	1	
Contraceptive use	No	19	24	25	26	94	0.686
	Yes	11	14	9	10	44	
Menstruation last 7 days	No	23	27	21	29	100	0.329
	Yes	7	11	13	7	38	
Education	Elementary School	0	0	1	1	2	0.182
	High School	23	24	26	37	110	
	Max. 3 years of higher education	15	21	18	17	79	
	4 years or more of higher education	32	29	0	25	104	
	PhD	2	0	1	2	5	
	Other	3	1	1	0	5	
Currently smoking	No	62	60	55	71	248	0.336
	Yes	18	15	18	11	57	

The four feedback-type groups did not differ in the ratings and psychological questionnaires either – see Table 2 for an overview. On average, participants rated the feedback stimuli as rather rewarding (M = 5.94, SD = 2.31). Please note that the first 18 participants in the star feedback group did not provide this rating because the question was only implemented later. Ratings of each feedback-type group were significantly higher than 5, the response alternative in the middle of the nine-point Likert scale (*t* test against the score 5; all *p* values < .008; all d’s > 2.0). This suggests that all four feedback stimuli were perceived as rewarding. Reward ratings did not differ for the four types of feedback (F(3,283) = 0.74, *p* = .532; BF_01_ = 23.83), see Table 2, “Reward rating”. At the end of the experiment, participants were informed about how often they had received positive feedback, which was on average 106.82 times (SD = 17.43). Again, no differences between feedback types were observed (F(3,301) = 0.39, *p* = .762; BF_01_ = 41.03). The participants seemed to have been aware of the skewed reward contingencies, as 83.3% of all participants correctly identified the stimulus that was rewarded more often (11.5% were undecided, 5.2% gave an incorrect response).

**Table 2:** Ratings and psychological questionnaires

		Star		Verbal FB		Happy face		Thumbs up		One-way ANOVA
		M	SD	M	SD	M	SD	M	SD	*p*
Willingness to pay for food (in EUR)		10.95	9.17	9.53	9.38	7.77	6.03	8.74	6.04	0.087
PANAS	Positive affect	30.04	6.42	28.77	7.80	28.19	8.01	28.11	7.30	0.348
	Negative affect	16.89	6.52	16.89	7.79	17.10	6.89	15.90	5.95	0.684
TEPS	Anticipatory	4.16	0.65	4.27	0.73	4.23	0.75	4.06	0.76	0.284
	Consummatory	4.41	0.67	4.60	0.70	4.55	0.73	4.42	0.74	0.241
BAQ		79.07	13.38	79.15	16.76	77.51	16.93	74.83	16.80	0.290
SHAPS		29.03	4.95	28.55	6.64	28.40	5.78	28.05	5.22	0.755
Final reward count PRT		105.20	19.03	106.59	18.04	108.19	17.17	107.28	15.66	0.762
Reward rating		6.02	2.06	5.77	2.44	5.74	2.36	6.22	2.30	0.532

#### Pre-registered results

##### Classical PRT analyses results (see Table 3, untransformed values for reaction time and accuracy rates)

Single-trial reaction time data (logarithmized) were faster for the rich than the lean stimulus (b = – 0.015, SE = 0.002, 95% CI [– 0.019; – 0.012], *t*(299) = – 7.86, *p* < .001, semi-partial *R*^2^ = 0.17). Reaction times were faster in block 2 than in block 1 (b = – 0.045, SE = 0.005, 95% CI [– 0.050; – 0.036], t(897) = – 9.14, p < .001, semi-partial R^2^ = 0.07), while differences between blocks 3 and 2 were not significant (p = .322). Moreover, reaction times became faster with increasing trial number (b = – 2.13e-4, SE = 1.98e-5, 95% CI [– 2.52e-4; – 1.75e-4], t(78047) = – 10.79, *p* < .001, semi-partial *R*^2^ = 0.002), with a very small effect size. Indicated by a significant interaction of trial number and block, this acceleration in reaction times was particularly obvious from block 1 to block 2 (b = – 7.07e-4, SE = 4.83e-5, 95% CI [– 8.02e-4; – 6.12e-4], *t*(78017) = – 14.64, *p* < .001, semi-partial *R*^2^ = 0.003), while reaction times became slower again from block 2 to block 3 (b = 1.16e-4, SE = 4.85e-5, 95% CI [2.04e-5; 2.11e-4], *t*(78021) = 2.38, *p* = .017, semi-partial *R*^2^ = 0.003). No other effects were significant (all *p* values > .316). A BF_01_ = 11.62 suggested strong evidence for the absence of differences between the feedback types.

**Table 3:** Reaction times, accuracy rates and signal detection indices

			Star		Verbal FB		Happy face		Thumbs up	
			M	SD	M	SD	M	SD	M	SD
RTs (sec)	Rich	Block1	0.60	0.16	0.59	0.12	0.58	0.11	0.60	0.16
		Block2	0.57	0.18	0.59	0.14	0.55	0.12	0.58	0.14
		Block3	0.57	0.17	0.59	0.18	0.53	0.10	0.59	0.16
	Lean	Block1	0.61	0.16	0.62	0.14	0.59	0.11	0.61	0.14
		Block2	0.60	0.20	0.61	0.15	0.57	0.11	0.60	0.13
		Block3	0.60	0.19	0.61	0.14	0.55	0.10	0.60	0.14
ACC (%)	Rich	Block1	74.13	15.59	75.99	14.10	76.44	16.33	76.22	13.05
		Block2	75.42	18.41	76.94	15.97	78.63	14.43	78.94	14.79
		Block3	78.22	16.45	77.69	18.44	79.46	16.48	77.87	14.93
	Lean	Block1	68.94	14.70	69.33	14.99	71.08	15.98	69.17	16.27
		Block2	67.20	18.60	69.34	18.15	70.85	14.97	70.02	14.46
		Block3	66.63	21.50	69.73	17.70	71.07	16.52	69.42	15.53
log b		Block1	0.07	0.23	0.08	0.22	0.07	0.28	0.08	0.22
		Block2	0.11	0.27	0.11	0.27	0.10	0.27	0.12	0.23
		Block3	0.14	0.25	0.13	0.31	0.13	0.30	0.11	0.22
log b reward learning			0.07	0.26	0.05	0.32	0.06	0.28	0.04	0.24
log d		Block1	0.45	0.28	0.47	0.28	0.52	0.28	0.47	0.26
		Block2	0.48	0.35	0.52	0.35	0.54	0.26	0.53	0.30
		Block3	0.49	0.35	0.54	0.34	0.58	0.33	0.51	0.31

Accuracy rates (after arcsine transformation) were significantly higher for the rich compared to the lean stimulus (b = 0.192, SE = 0.024, 95% CI [0.144; 0.240], *t*(301) = 7.91, *p* < .001, semi-partial *R*^2^ = 0.17) and higher in block 2 than in block 1 (b = – 0.027, SE = 0.014, 95% CI [– 0.054; – 1.44e-4], *t*(1026) = – 1.97, *p* = .049, semi-partial *R*^2^ = 0.01). Moreover, the accuracy difference between the rich and the lean stimulus was larger in block 2 than in block 1 (b = – 0.054, SE = 0.027, 95% CI [– 0.107; – 9.43e-4], t(904) = – 1.99, *p* = .046, semi-partial *R*^2^ = 0.01). No other effects were significant (all *p* values > .148). A BF_01_ = 26.93 suggested strong evidence for the absence of differences between the feedback types.

Reward sensitivity assessed via log b was significantly higher in block 2 than in block 1 (b = – 0.04, SE = 0.01, 95% CI [– 0.07; – 0.01], *t*(602) = – 2.67, *p* = .008, semi-partial *R*^2^ = 0.02), while no significant differences were found between blocks 2 and 3 (*p* = .273). Neither feedback-type nor the interaction effects were significant (all *p* values > .410). A BF_01_ = 65.44 suggested very strong evidence for the absence of differences between the feedback types.

Stimulus discriminability assessed via log d was significantly higher in block 2 than in block 1 (b = – 0.04, SE = 0.01, 95% CI [– 0.06; – 0.01], *t*(602) = – 3.02, *p* = .003, semi-partial *R*^2^ = 0.03), while no significant differences were found between blocks 2 and 3 (*p* = .319). Neither feedback-type nor the interaction effects were significant (all *p* values > .122). A BF_01_ = 23.93 suggested strong evidence for the absence of differences between the feedback types.

##### Drift diffusion model results

**Fig. 2:**
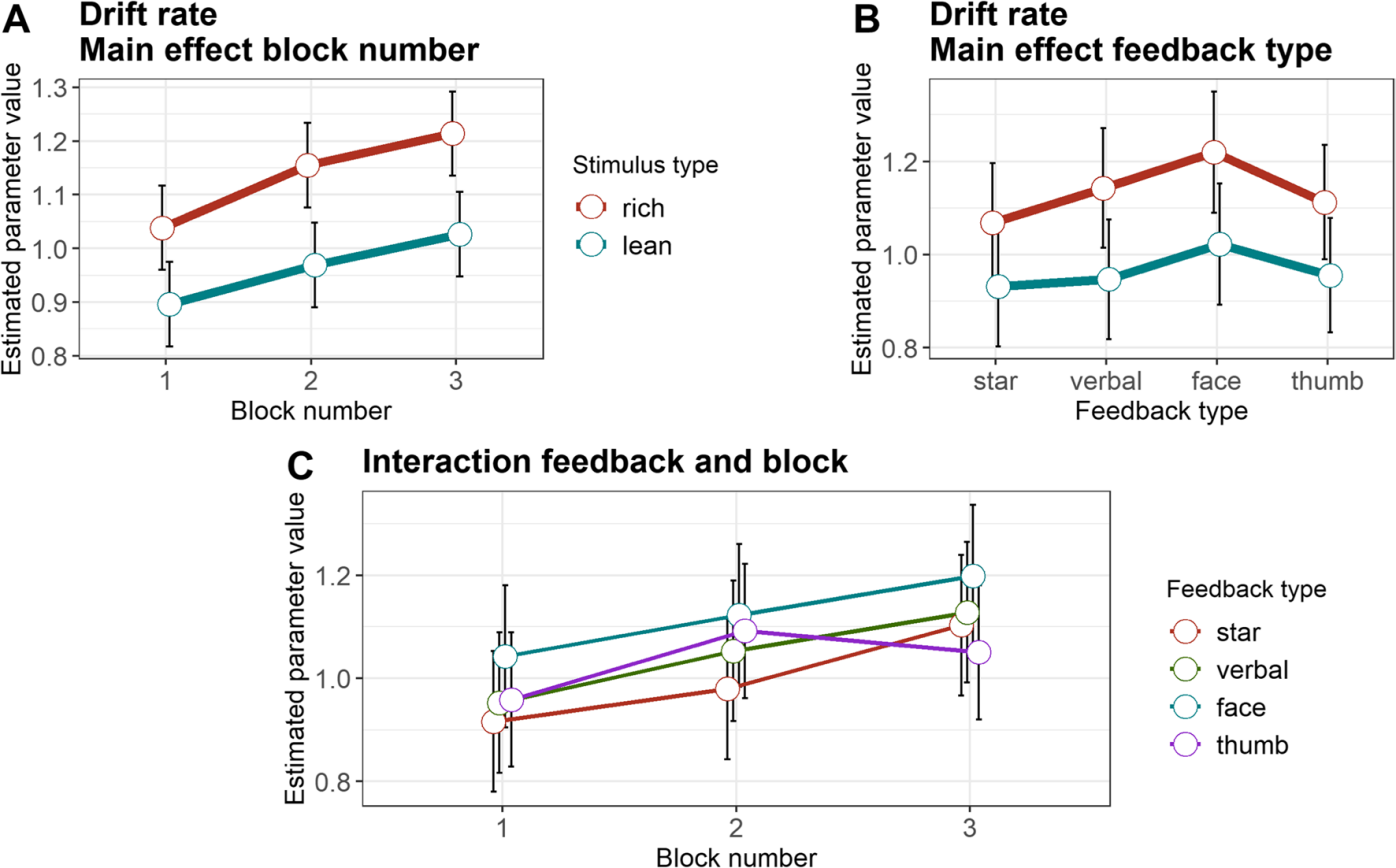
Drift rate parameter. **A** Estimated parameter values, averaged across feedback types. **B** Estimated parameter values, averaged across blocks. **C** Estimated parameter values, averaged across reward contingencies. *Error bars* depict the standard error of the mean

Efficiency in evidence accumulation, as reflected in drift rates (*v*, Fig. 2, Table 4), was higher for the rich than the lean stimulus (b = – 0.17, SE = 0.03, 95% CI [– 0.23; – 0.11], *t*(301) = – 5.32, *p* < .001, semi-partial *R*^2^ = 0.09). Moreover, drift rates increased from block 1 to block 2 (b = – 0.10, SE = 0.02, 95% CI [– 0.14; – 0.06], *t*(1204) = – 4.66, *p* < .001, semi-partial *R*^2^ = 0.05), and from block 2 to block 3 (b = – 0.06, SE = 0.02, 95% CI [– 0.10; – 0.02], t(1204) = – 2.78, *p* = .006, semi-partial *R*^2^ = 0.05). These findings are in line with previous research (Eikemo et al., 2019). Our analyses further showed a significant interaction between feedback-type and block when comparing blocks 2 and 3 for the star and the thumbs-up feedback, with a small effect size (b = 0.14, SE = 0.06, 95% CI [0.03; 0.25], *t*(1204) = 2.49, *p* = .013, semi-partial *R*^2^ = 0.01). Descriptively resolving this interaction, the drift rates increased linearly from the first to the last block with the star feedback. In contrast, the drift rates increased from block 1 to 2 with the thumbs-up feedback, while drift rates in block 3 where lower than in block 2. No other effects were significant (all *p* values > .153).

**Table 4:** Drift diffusion model parameters

			Star		Verbal FB		Happy face		Thumbs up	
			M	SD	M	SD	M	SD	M	SD
Drift rate	Rich	Block 1	0.99	0.59	1.09	0.56	1.15	0.64	1.03	0.56
		Block 2	1.11	0.64	1.16	0.67	1.22	0.63	1.24	0.65
		Block 3	1.22	0.70	1.25	0.78	1.35	0.69	1.13	0.63
	Lean	Block 1	0.91	0.56	0.86	0.50	0.99	0.57	0.93	0.60
		Block 2	0.95	0.65	1.00	0.63	1.05	0.54	0.97	0.59
		Block 3	1.07	0.74	1.05	0.63	1.09	0.59	1.02	0.53
Boundary separation	Rich	Block 1	1.30	0.23	1.29	0.20	1.29	0.16	1.30	0.19
		Block 2	1.26	0.27	1.29	0.20	1.23	0.18	1.28	0.17
		Block 3	1.27	0.23	1.31	0.22	1.22	0.17	1.29	0.20
	Lean	Block 1	1.29	0.21	1.30	0.20	1.28	0.18	1.30	0.18
		Block 2	1.24	0.26	1.28	0.16	1.22	0.19	1.26	0.17
		Block 3	1.27	0.23	1.29	0.18	1.20	0.17	1.27	0.19
Non-decision time	Rich	Block 1	0.89	0.06	0.91	0.04	0.91	0.04	0.91	0.04
		Block 2	0.90	0.05	0.91	0.05	0.92	0.03	0.90	0.04
		Block 3	0.90	0.05	0.91	0.04	0.93	0.03	0.90	0.05
	Lean	Block 1	0.89	0.05	0.91	0.04	0.92	0.03	0.90	0.04
		Block 2	0.90	0.05	0.91	0.05	0.92	0.04	0.90	0.05
		Block 3	0.90	0.05	0.91	0.04	0.93	0.03	0.91	0.04
Starting bias		Block 1	0.52	0.04	0.51	0.05	0.52	0.05	0.52	0.05
		Block 2	0.52	0.06	0.52	0.05	0.53	0.06	0.53	0.06
		Block 3	0.54	0.06	0.53	0.06	0.53	0.05	0.53	0.05

**Fig. 3:**
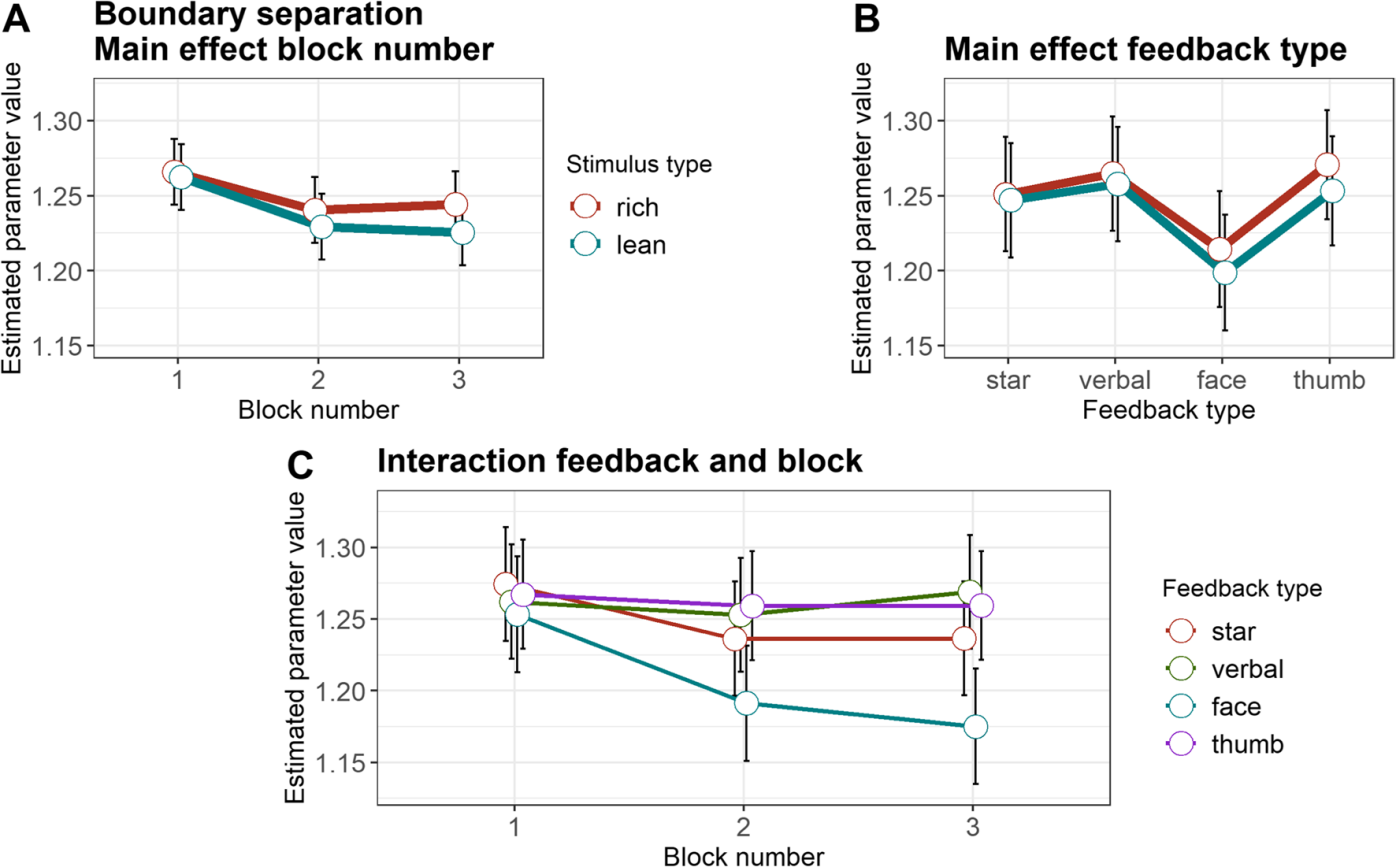
Boundary separation. **A** Estimated parameter values, averaged across feedback types. **B** Estimated parameter values, averaged across blocks. **C** Estimated parameter values, averaged across reward contingencies. *Error bars* depict the standard error of the mean

The amount of evidence necessary for a choice, as reflected in the boundary separation parameter (*a*, Fig. 3, Table 4), was higher for the rich than the lean stimulus (b = – 0.01, SE = 0.004, 95% CI [– 0.017; – 0.009], *t*(301) = – 2.17, *p* = .031, semi-partial *R*^2^ = 0.02). Moreover, there was an overall decrease in necessary evidence from block 1 to block 2 (b = 0.04, SE = 0.009, 95% CI [0.02; 0.06], *t*(301) = 4.21, *p* < .001, semi-partial *R*^2^ = 0.06). This main effect of block was further qualified by a significant interaction between feedback type and block when comparing blocks 2 and 3 for the star and the happy face feedback (b = 0.05, SE = 0.21, 95% CI [0.006; 0.088], *t*(301) = 2.22, *p* = .027, semi-partial *R*^2^ = 0.03). Descriptively resolving this interaction, the boundary separation parameter was smaller in block 2 than block 3 with the star feedback, whereas the pattern was reversed with the happy face, i.e., a smaller boundary separation parameter in block 3 than block 2. This suggests that the responses of the happy face feedback group were faster, but more error-prone in block 3 than in block 2, while the responses of the star feedback group were faster, but more error-prone in block 2 than in block 3.

**Fig. 4:**
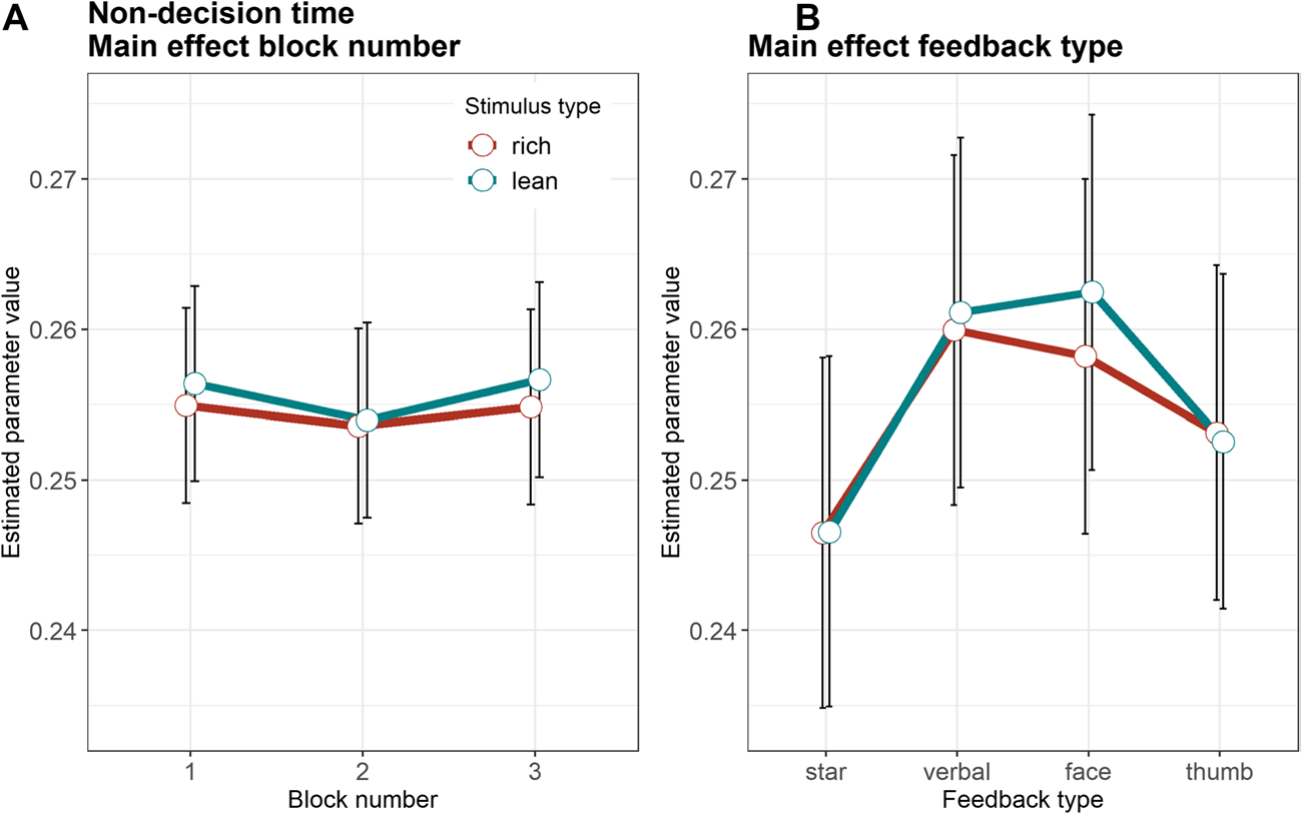
Non-decision time. **A** Estimated parameter values, averaged across feedback types. **B** Estimated parameter values, averaged across blocks. *Error bars* depict the standard error of the mean

The time between stimulus onset and evidence accumulation, as reflected in the non-decision time parameter (*t*, Fig. 4, Table 4) showed significant differences between the star and the verbal feedback (b = 0.02, SE = 0.005, 95% CI [0.004; 0.025], *t*(301) = 2.66, *p* = .008, semi-partial *R*^2^ = 0.08), and between the star and the happy face feedback (b = 0.03, SE = 0.006, 95% CI [0.02; 0.04], *t*(301) = 4.94, *p* < .001, semi-partial *R*^2^ = 0.08). The non-decision time was shorter with the star feedback than the two other feedback types. No other effects were significant (all *p* values < .090).

**Fig. 5:**
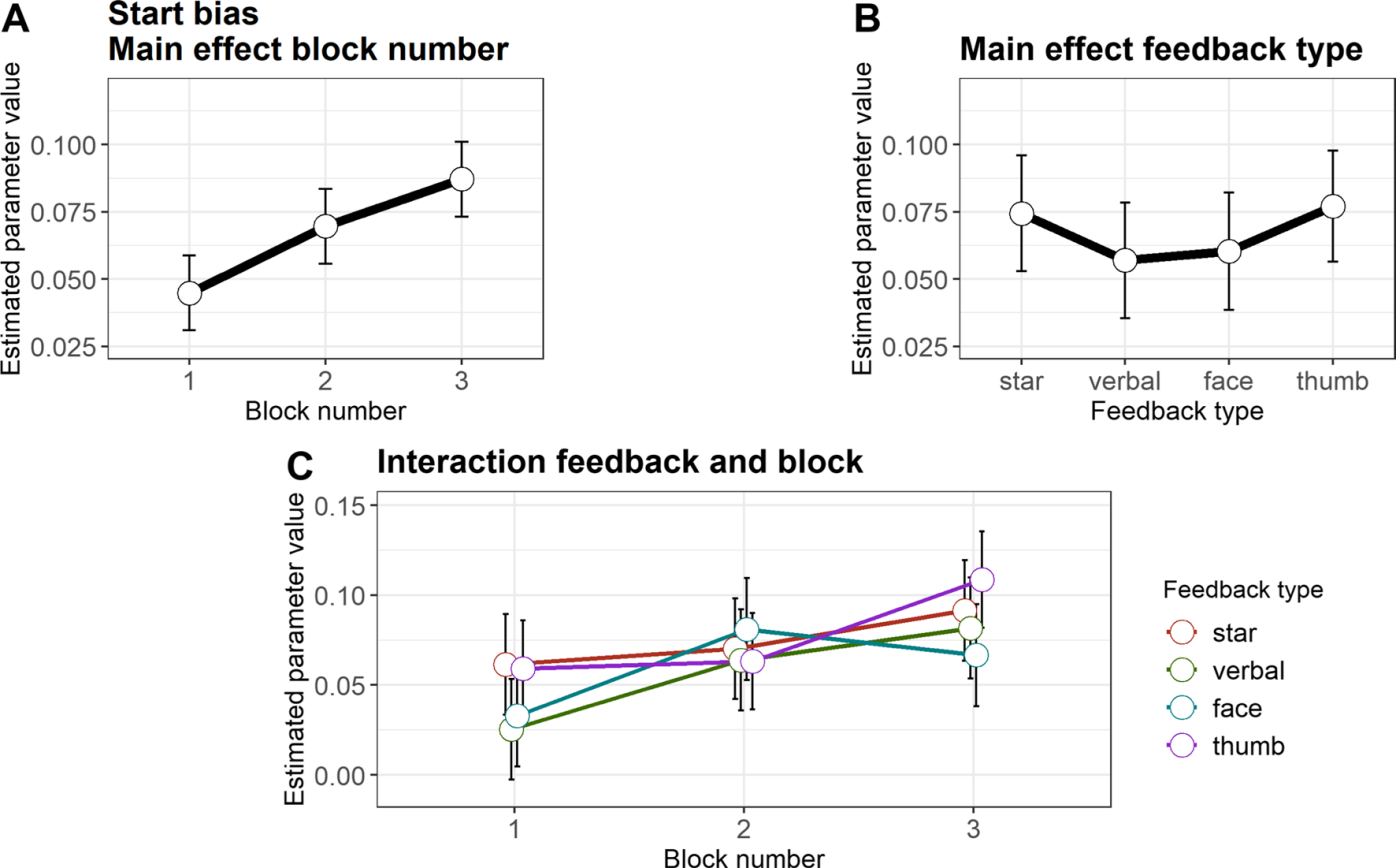
Starting bias. **A** Estimated parameter values for blocks, averaged across feedback types and reward contingency. **B** Estimated parameter values for feedback type, averaged across block and reward contingency. **C** Estimated parameter values, averaged across reward contingency. *Error bars* depict the standard error of the mean

In line with previous findings, we found a bias towards choices for the rich stimulus option, as reflected in the starting point parameter (*z*, Fig. 5, Table 4). The estimated starting bias across participants (collapsed over blocks and reward contingency, M = 0.53, SD = 0.04) was significantly different from 0.5, *t*(304) = 12.08, *p* < .001, d = 0.69, 95% CI [0.57; 0.82]. The starting bias increased from block 1 to block 2 (b = – 0.01, SE = 0.003, 95% CI [– 0.015; – 0.004], t(602) = – 3.21, *p* = .001, semi-partial R^2^ = 0.06), and from block 2 to block 3 (b = – 0.008, SE = 0.003, 95% CI [– 0.014; – 0.002], *t*(602) = – 2.69, *p* = .007, semi-partial *R*^2^ = 0.06). Moreover, a significant interaction effect was observed when comparing blocks 2 and 3 for the star and the happy face feedback (b = 0.05, SE = 0.21, 95% CI [0.009; 0.004], *t*(301) = 2.22, *p* = .027, semi-partial *R*^2^ = 0.02). Descriptively resolving the interaction, the starting bias was larger in block 3 than in block 2 with the star feedback, while the starting bias was larger in block 2 than in block 3 with the happy face feedback. This suggests that the preference for the rich stimulus in the final experimental block was smaller with the happy face feedback.

#### Exploratory results

Reward learning, i.e., the difference in log b between blocks 3 and 1, differed significantly from zero in the total sample (t(304) = 3.36, *p* < .001, d = 0.19, CI [0.08; 0.31]). No differences were observed in reward learning for the four feedback types though (*F*(3,301) = 0.16, *p* = .920). A BF_01_ = 54.64 suggested very strong evidence for the absence of differences between feedback types in reward learning.

Adding individual scores of trait reward responsiveness (TEPS) or current positive and negative affective states (PANAS) as predictors for log b (i.e., participants’ response bias towards the more often rewarded option) did not result in significant effects of these variables (all *p* values > .319). In contrast, hedonic capacity (SHAPS) interacted with differences between the non-social group (star) and the social group presented with thumbs up (b = – 0.018, SE = 0.007, *t*(297) = – 2.76, *p* = .006, semi-partial *R*^2^ = 0.03). The star feedback group showed a positive association between log b and hedonic capacity (simple effects analysis: b = 0.011, SE = 0.005, 95% CI [0.001; 0.021], *t*(297) = 2.18, *p* = .030), while the thumbs up feedback group showed a non-significant negative association (b = – 0.008, SE = 0.005, 95% CI [– 0.016; 0.001], *t*(297) = – 1.70, *p* = .091). Participants’ sex (both *p* values > .673) and age (both *p* values > .031) had no influence on log b variation. Psychological questionnaires, participants’ sex and age had no impact on the DDM parameters starting bias (all *p* values > .090), boundary separation (all *p* values > .036), and drift rate (all *p* values > .064). For the DDM parameter non-decision time, a significant interaction between participants’ age and the star and the thumbs up feedback group was observed (b = – 0.003, SE = 6.95e-4, 95% CI [– 0.004; – 0.002], *t*(297) = – 4.18, *p* < .001, semi-partial *R*^2^ = 0.06). The star feedback group showed a positive association between age and non-decision time (simple effects analysis: b = 0.002, SE = 5.45e-4, 95% CI [8.01e-4; 0.003], *t*(297) = 3.44, *p* < .001), while the thumbs up feedback group showed a negative association (simple effects analysis: b = – 0.001, SE = 4.42e-4, 95% CI [– 0.002; – 1.64e-4], *t*(297) = – 2.34, *p* = .020).

## Discussion

This pre-registered online experiment aimed to identify underlying mechanisms for a proposed processing advantage of social over non-social feedback stimuli. In particular reward learning (behavioural preference for a rewarded choice option) and sub-processes influencing reward responsivity (facilitation of evidence accumulation or a general bias towards the rewarded option) were investigated. While the online administration of an established probabilistic reward task (PRT, Pizzagalli et al., 2005) led to the expected response bias towards the more frequently rewarded response option, the hypothesised processing advantage of social over non-social feedback stimuli was not observed. In contrast, almost no evidence for processing differences between social and non-social feedback stimuli in the PRT was found.

A strength of the current PRT study is that the calculation of participants’ response bias (log b) and discriminability bias (log d) was complemented by a computational modelling approach (Barch et al., 2017; Huys et al., 2013; Ratcliff et al., 2016), which allowed for a more fine-grained assessment of sub-processes of reward responsivity. The results demonstrated the validity of the online administration of the current PRT. Participants chose the rewarded option (the “rich” stimulus) more often, and they were faster and more accurate when doing so. This resulted in stable response (log b) and discriminability (log d) biases in line with previous research (Pizzagalli et al., 2005). The drift diffusion modelling showed better evidence accumulation for the rich stimulus (drift rate) and a higher starting bias for the rich stimulus, in line with Huys et al. (2013). However, contrary to our hypothesis, we observed no evidence for a processing advantage of social over non-social feedback stimuli in these two modelling parameters. Moreover, no group differences were observed in reaction times, accuracy rates, the signal detection indices, and reward learning between the different groups applying either social or the standard non-social feedback stimuli. The only group difference was observed for the non-decision time modelling parameter, which was not a priori in the focus. Verbal and happy face feedback stimuli led to a slight delay in the evidence accumulation process compared to the star feedback stimulus. However, overall performance of the four feedback groups was comparable, which renders the impact of the non-decision time result negligible.

The current findings further suggest that the previously reported processing advantage of social over non-social stimuli (Hurlemann et al., 2010; Pfabigan et al., 2019; Pfabigan & Han, 2019) does not apply to a between-subjects set-up in which one category of rewarding stimuli (equivalent to positive performance feedback) was presented in a probabilistic way. The application of a between-subjects design is one of the main differences between the current study and previous within-subject studies reporting a processing advantage. In these, participants were confronted with both social and non-social stimuli within the same experiment. The continuous confrontation with both social and non-social performance feedback could have led to direct comparison processes between the two, which might have led to the observed processing advantage of social over non-social stimuli. It is thus possible that the direct comparison of social with non-social stimuli is necessary to establish the observed processing advantage. For example, non-social stimuli could serve as anchors for the subsequent assessment of social stimuli, or vice versa (e.g., Meyer & Schvaneveldt, 1971; Tversky & Kahneman, 1974).

Similar reasoning as for a potential processing benefit applies to the evaluation of subjective reward value. Although the four feedback stimuli were rated as similarly rewarding, it is important to keep in mind that these ratings were obtained by different participants. Thus, the participants presumably compared the feedback they received to no feedback at all. The reward ratings might look differently if the same participants rated all four feedback types. Along these lines, ratings of touch as a social stimulus were found to differ depending on whether a between- or a within-subjects design was used (Triscoli et al., 2014). In this study, different velocities of stroking were rated more similarly to each other when they were rated by different participants (between-subjects design) than when they were rated by the same participants (within-subjects design). In a different domain, signal intensity elicited greater effects on conditioning and reaction time in a within- than between-subjects design (Grice & Hunter, 1964). Based on these findings, one may speculate that a within-subjects design might have led to larger differences between the four feedback types. However, for the present study, we were interested in the methodical aspects of the PRT, i.e., if the PRT elicits different behavioural preferences depending on the feedback type, and in this task there is typically only one type of feedback presented. Applied to a more general learning context such as finding the optimal type of feedback for a language learning app, the absolute effectiveness of a particular feedback would also be more important than its relative effectiveness. For these reasons, we opted for a between-subjects design.

Another explanation for the absence of the hypothesised processing advantage could be the use of only behavioural instead of neural outcomes. Assessing the Feedback-Related Negativity component FRN (Gehring & Willoughby, 2002; Miltner et al., 1997), a neural marker of feedback processing, changes in response bias from blocks 2 and 3 compared to block 1 were positively related to FRN amplitude variation (Santesso et al., 2008). However, this positive relationship was not observed for actual task behaviour. Another study reported an association between a PRT reward bias and neural markers even when FRN amplitudes were assessed in an independent task (Bress & Hajcak, 2013). Similarly, a processing advantage of social over non-social feedback stimuli was demonstrated in FRN amplitude variation in our previous studies (Pfabigan et al., 2019; Pfabigan & Han, 2019), whereas differences in behaviour were either absent or only small. Therefore, it could be possible that neural markers of PRT performance are more sensitive to a processing advantage of social over non-social stimuli than behavioural markers. On a more general note, the utilization of neurophysiological markers of decision-making could advance our understanding of the underlying processes captured by the task. For example, event-related activity on a trial basis can be reliably linked to drift diffusion modelling processes and parameters such as evidence accumulation, drift rate, or boundary stopping criteria (Kelly & O’Connell, 2013), which could be of particular interest when aiming to replicate the boundary separation effect observed in the happy face feedback group. Furthermore, neural information allows valuable insight to arbitrate and empirically test different evidence-accumulation models beyond the possibilities of conventional behavioural measures (Devine et al., 2019; Kelly et al., 2021).

On a conceptual level, one might also ask whether the current operationalisation of social and non-social reward stimuli was optimal. The social stimuli used (praise indicated by a speech bubble, a smiling face, and thumbs up) might not have been very social after all because no interaction with a human occurred. In contrast, the non-social stimulus (a yellow star with the text “Correct! You won 1 point!”) might have been perceived as social, for example, if participants assumed that this feedback came from the experimenter. This type of feedback was selected to allow comparability with most published PRT studies using a similar stimulus. Because it primarily conveys information about task performance (“Correct!”) and potential reward (points in the current study, but most often monetary reward in other studies), we considered it to reflect rather non-social reward provided by the computer program. In contrast, the social feedback stimuli of the current study were selected because their physical stimulus features were indicative of critical aspects of social interactions – for example the communication of social motives such as affiliation and protection via a smiling face or of approval via the thumbs up gesture (Morris, 1994).

What makes a stimulus social is an ongoing debate. In previous studies, we argued that physical stimulus features linked to social interactions (i.e., gestures, facial expressions) are enough to render a stimulus social (Pfabigan et al., 2019; Pfabigan & Han, 2019). In contrast, other authors argued that it is the social context in which the stimuli are embedded that renders them social, for example via the construction of ingenious cover stories where participants were led to believe to receive feedback from other individuals (Kujawa et al., 2014; Somerville et al., 2006). These diverging definitions of social stimuli – based on a bottom-up or a top-down definition – could serve as a forward-looking basis for further studies. For example, an experiment could compare feedback information via non-social feedback stimuli that have either an assigned social connotation (i.e., feedback stimuli selected by the participant’s partner) or not (i.e., feedback stimuli generated by a computer program).

The current results provide additional future-oriented insights into the application of the PRT. While online experiments are thought to be susceptible to potential differences in hardware, operating systems, the chosen browser application, and the respective situation in which participants conduct the experiment (see Bridges et al., 2020; Semmelmann & Weigelt, 2017), recent studies have established their validity also for cognitive experiments. For example, Semmelmann & Weigelt (2017) tested five experimental tasks both in a controlled laboratory setting and in a less-controlled web setting, and concluded that the relevant task-specific effects could be replicated (apart from a priming experiment) and that error rates were comparable in both settings. A recent mega-study further corroborated the timing precision of online experiments and emphasised the usability of online experiments in experimental research (Bridges et al., 2020). Thus, the current results demonstrate that an online administration of the PRT results in the expected effects without much loss of experimental control. The only discrepancy with PRT laboratory assessments compared to the current online application were the responses to the post-experimental question as to whether or not participants were aware of the skewed reward scheme. The majority of the current participants seemed to have been aware of it, while this is most often not the case in laboratory settings (Eikemo et al., 2017; Heerey et al., 2008). Nevertheless, we refrain from regarding this knowledge as a disadvantage of the online application because the current participants nevertheless displayed the expected response bias.

Moreover, in contrast to the classical laboratory settings in which participation is incentivised with a monetary bonus for each received reward, the current online PRT assessment used a different strategy. Participants knew beforehand how much money they would be compensated with, irrespective of their performance. This suggests that omitting a direct link between received rewards and monetary outcomes still yielded the expected response preference for the highly rewarded response option. This underlines the robustness of the PRT. Online versions of the PRT could facilitate its application in difficult-to-reach settings (e.g., forensics, marginalized participant groups, sparsely populated areas) and allow for higher feasibility of longitudinal designs.

In an exploratory analysis, we tested whether PRT outcomes were associated with self-reported reward responsiveness. Of particular interest was the correlation between reward responsivity assessed with log b and hedonic capacity assessed with the SHAPS (Franken et al., 2007; Snaith et al., 1995). Previous studies have reported a negative association between those two, which was primarily demonstrated in clinical samples (Luking et al., 2017; Pizzagalli et al., 2005; Vrieze et al., 2013) and their first-degree relatives (Liu et al., 2016). This association was not present in our overall sample (*r* = 0.05; BF_01_ = 9.86, suggesting moderate evidence for the absence of an association between hedonic capacity and reward responsivity). However, an exploratory analysis showed opposing associations between the reward bias and hedonic capacity in the groups presented with the non-social (star) and a social feedback stimulus (thumbs up), whereas the groups did not differ overall in hedonic capacity. Future research should examine whether this exploratory finding indicates a flexible and malleable relationship between state and trait measures of reward responsivity. The DDM parameters were also not associated with self-reported hedonic capacity. Of note, the current online sample had on average hedonic capacity scores that were higher than those reported for healthy individuals in a recent meta-analysis (Trøstheim et al., 2020). Thus, the question whether hedonic capacity influences the processing of social and non-social feedback stimuli cannot be conclusively answered.

## Conclusion

No processing advantage of social over non-social feedback stimuli was observed when they were presented to different participants. Overall, these findings strongly suggest that different social stimuli can serve equally well as reinforcing stimuli in the probabilistic reward task (PRT), without affecting the expected response bias towards the more frequently rewarded option.

## Data Availability

The study was pre-registered on the Open Science Forum (https://osf.io/24qtv). All data and materials are available via the Open Science Framework: https://osf.io/nu3hm/?view_only=72cc25927eda459ca7615cd4b8ba1141 (view-only link for peer review) and https://github.com/fwurm/PRT_ddm_online. This manuscript was deposited as a preprint on the server under a CC-BY-NC-ND 4.0 International license: https://psyarxiv.com/zm6gj/

## Code availability

Via the Open Science Framework and GitHub: https://osf.io/nu3hm/?view_only=72cc25927eda459ca7615cd4b8ba1141

(view-only link for peer review), https://github.com/fwurm/PRT_ddm_online
